# TRAJECTORIES OF LONELINESS IN LATER LIFE: EVIDENCE FROM A 10-YEAR ENGLISH PANEL STUDY

**DOI:** 10.1093/geroni/igae098.0287

**Published:** 2024-12-31

**Authors:** Giorgio Di Gessa

**Affiliations:** University College London, London, England, United Kingdom

## Abstract

Loneliness is generally defined as the discrepancy between individuals’ desired and actual social interactions and emotional support. Although the prevalence of loneliness is high among older people and is projected to rise, few studies have examined longitudinal patterns of loneliness. Moreover, most studies have focused on more “objective” risk factors for loneliness such as partnership status and frequency of contact, overlooking the quality of the relationship with and support from family and friends. Using data from six waves of the English Longitudinal Study of Ageing (2008/09 to 2018/19, N=4740), we used group-based trajectory modelling to identify distinctive trajectories of loneliness. Multinomial regression models were then used to examine characteristics associated with these trajectories, with a particular focus on size, support, closeness, and frequency of contact with social network members. We identified 5 groups of loneliness trajectories in later life, representing “stable low” (40% of the sample), “medium/low” (26%), “stable high” (11%), and “increasing” (14%) or “decreasing” (9%) levels of loneliness over time. Although there are socioeconomic and demographic differences across these trajectories of loneliness, health and relationship quality are their main drivers. Respondents with poor and deteriorating health were more likely to be classified as having “stable high” or “increasing” loneliness. Even if not having social networks is undoubtedly associated with higher risks of persistent loneliness, having friends and family is not enough: Respondents with low-quality relationships with both friends and family were also significantly more likely to be classified as having “stable high” or “increasing” levels of loneliness.

